# B’ACTIV Behavioral Activation, Nutrition, and Activity Intervention for Persons Aging With HIV

**DOI:** 10.1093/geroni/igaf122.944

**Published:** 2025-12-31

**Authors:** Ann Gruber-Baldini, Laura Nnadi, Renee Pepin, Elizabeth Dennis, Nancy Latham, Denise Orwig

**Affiliations:** University of Maryland School of Medicine, Baltimore, Maryland, United States; University of Maryland School of Medicine, Baltimore, Maryland, United States; Geisel School of Medicine at Dartmouth, Lebanon, New Hampshire, United States; University of Maryland School of Medicine, Baltimore, Maryland, United States; Brigham and Women’s Hospital, Boston, Massachusetts, United States; University of Maryland, Baltimore, Baltimore, Maryland, United States

## Abstract

B’ACTIV is a pilot study of a 12-week Behavioral Activation intervention targeted to decrease loneliness and increase social engagement, that was augmented with specific content to increase home exercise and address poor nutrition among persons aging with HIV (PAWH). The BA intervention includes a coach that focuses participants on their values to align them with behaviors to increase engagement. Recruitment included those ages 50+ with HIV from the University of Maryland THRIVE program. This work has been completed in three stages: Stage 1: A community engagement team of 5 PAWH provided feedback on the intervention, including preferences for delivery mode, recruitment strategies and brochures, measures, other study materials, and inclusion of the physical activity and nutrition modules. Recommendations included use of telephone/in-person intervention, deletion of “HIV” in materials to avoid stigma, and inclusion of foods to help with medication side effects of nausea, lack of appetite, and difficulty swallowing. Stage 2: We worked with HIV clinical and research experts in exercise, nutrition, and psychological functioning to tailor the content of the material and resources for the coaches to meet the needs of PAWH. Stage 3: Following the community engagement and tailoring the intervention for PAWH, we tested the feasibility and acceptability of the intervention. There are currently 7 enrolled (5 active, 1 completed, 1 withdrew). Mean age is 60.0 (SD = 7.5, range 51-75); 5 females/2 males; 6 AA/1 white. This study tests the feasibility of a much-needed intervention to improve functioning and health outcomes among a vulnerable group of older adults.

